# Supplementary material for: First quantitative high-throughput screen in zebrafish identifies novel pathways for increasing pancreatic β-cell mass
Source: eLife. 2015 Jul 28;4:e08261. doi: 10.7554/eLife.08261 (PMC4534842; doi:10.7554/eLife.08261)
Supplement: Supplementary file 1. — ARQiv Hit calls. The 46 compounds implicated as Hit Calls following the ARQiv screen and initial visual assessments of enhanced 2° islet formation are listed. Compounds are ordered according to SSMD value. Hit I and Hit II subsets are indicated by a check mark in the corresponding column. In addition, Hit I compounds that were evaluated in β-cell proliferation assays alongside Hit II compounds are indicated as ‘tested’. Also listed are clinical indication, FDA approval status, or naming convention for compounds approved by FDA counterparts in other countries. DOI: http://dx.doi.org/10.7554/eLife.08261.018 [file elife08261s001.docx]

**Supplementary file 1. ARQiv Hit Calls (ordered by SSMD score)**

|  | **Drug Name** | **SSMD** | **Hit I** | **Hit II** | **Clinical indication** | **FDA** |
| --- | --- | --- | --- | --- | --- | --- |
| 1 | Paroxetine HCl | 3.15 |  | √ | Antidepressant | Yes |
| 2 | Promethazine HCl | 2.46 |  | √ | Antihistaminic | Yes |
| 3 | Pasiniazid | 2.32 |  | √ | Antibacterial | INN, DCF, MI |
| 4 | Benzalkonium Chloride | 2.31 |  | √ | Antiseptic (topical) | Yes |
| 5 | Amcinonide | 2.28 |  | √ | Glucocorticoid | Yes |
| 6 | NCS-382 | 2.12 |  | √ | Anticonvulsant | No |
| 7 | Chloroacetoxyquinoline | 2.09 |  | √ | Antifungal | No |
| 8 | Thioctic Acid (Lipoamide) | 2.07 | √ | tested | Hepatoprotectant | JAN |
| 9 | Acyclovir | 2.06 |  | √ | Antiviral | Yes |
| 10 | Phenothrin | 2.01 |  | √ | Insecticide | INN, BAN, MI |
| 11 | Diphenhydramine | 1.99 |  | √ | Antihistaminic | Yes |
| 12 | Reserpine | 1.95 |  | √ | Antihypertensive | Yes |
| 13 | RIAA 94 | 1.94 |  | √ | n/a | No |
| 14 | Trientine | 1.92 |  | √ | Antidote | Yes |
| 15 | Resorcinol Monoacetate | 1.91 |  | √ | Corneal trauma indicator | Yes |
| 16 | Beta-propiolactone | 1.90 |  | √ | Antiseptic | Yes |
| 17 | Khellin | 1.89 |  | √ | Vasodilator (coronary) | INN, DCF, MI |
| 18 | Butenafine HCl | 1.89 |  | √ | Antifungal | Yes |
| 19 | Emodic Acid | 1.85 |  | √ | Cathartic | No |
| 20 | Promazine HCl | 1.85 |  | √ | Antiemetic | Yes |
| 21 | Biperiden HCl | 1.83 | √ | tested | Antiparkinsonian. | Yes |
| 22 | Nomifensin Maleate | 1.82 |  | √ | Antidepressant | Yes |
| 23 | D-Gluconic acid calcium salt | 1.81 |  | √ | Nutrient- Ca2+ replenisher | Yes |
| 24 | Decitabine | 1.78 | √ | tested | Antineoplastic | INN, BAN |
| 25 | Amitriptyline | 1.75 |  | √ | Antidepressant | Yes |
| 26 | Ethopropazine HCl | 1.75 |  | v | Antiparkinsonian | Yes |
| 27 | N-Acetylaspartic Acid | 1.60 | √ | tested | Analeptic | No |
| 28 | Iodine | 1.56 | √ |  | Antiseptic | Yes |
| 29 | Methylthiouracil | 1.56 | √ |  | Thyroid | Yes |
| 30 | Parthenolide | 1.45 | √ |  | Anti-inflamation | No |
| 31 | Nevirapine | 1.45 | √ | tested | Antiviral | Yes |
| 32 | Dimethindene (S, +) maleate | 1.44 | √ |  | Antihistaminic | Yes |
| 33 | Thiram | 1.43 | √ | tested | Antiseptic | Yes |
| 34 | Maprotiline | 1.42 | √ |  | Antidepressant | Yes |
| 35 | N-Acetylmuramic acid | 1.40 | √ |  | n/a | No |
| 36 | Bayberry wax | 1.39 | √ |  | Therapeutic plant extract | Yes |
| 37 | Vitamin K3 | 1.38 | √ | tested | Vitamin | Yes |
| 38 | 1,5-Bis(succinimidooxycarbonyloxy)pentane | 1.37 | √ |  | n/a | No |
| 39 | BOC-S-acetaminomethyl-L-cysteine | 1.35 | √ | tested | n/a | No |
| 40 | Tretinoin | 1.34 | √ | tested | Antiacne | Yes |
| 41 | Ethynodiol diacetate | 1.33 | √ | tested | Contraceptive | Yes |
| 42 | Estradiol diacetate | 1.33 | √ |  | Estrogen | No |
| 43 | Benzamidine | 1.32 | √ |  | n/a | No |
| 44 | Oxacillin | 1.31 | √ |  | Antibiotic | Yes |
| 45 | Dexetimide | 1.30 | √ |  | Antiparkinsonian | INN, BAN |
| 46 | Fosphenytoin sodium | 1.30 | √ | tested | Anticonvulsant | Yes |

Abbreviations: INN: International Nonproprietary Names; DCF: Dénominations Communes Françaises; MI: Modified International Nonproprietary Names; BAN: British Approved Names; JAN: Japanese Adopted Names.

**Supplementary file 1. ARQiv Hit Calls**

The 46 compounds implicated as Hit Calls following the ARQiv screen and initial visual assessments of enhanced 2º islet formation are listed. Compounds are ordered according to SSMD value. Hit I and Hit II subsets are indicated by a check mark in the corresponding column. In addition, Hit I compounds that were evaluated in β-cell proliferation assays alongside Hit II compounds are indicated as "tested". Also listed are clinical indication, FDA approval status, or naming convention for compounds approved by FDA counterparts in other countries.
