# Supplementary material for: First quantitative high-throughput screen in zebrafish identifies novel pathways for increasing pancreatic β-cell mass
Source: eLife. 2015 Jul 28;4:e08261. doi: 10.7554/eLife.08261 (PMC4534842; doi:10.7554/eLife.08261)
Supplement: Supplementary file 2. — Outstanding Hit calls. The 131 compounds implicated in the primary screen but which remain to be further evaluated are listed here in subgroups according to their implicated pharmacological activity (shaded subsets). Subcategories included: neuromodulators, glucocorticoids, and retinoids—also implicated by Tsuji et al.,—as well as 11 other categories unique to our study. n/a: not applicable. DOI: http://dx.doi.org/10.7554/eLife.08261.019 [file elife08261s002.docx]

**Supplementary file 2. Outstanding Hit Calls**

| **Drug** | **Pharmacological Activity** |
| --- | --- |
| Quipazine maleate | Serotonin receptor agonist |
| Tranylcypromine HCl (trans-2-phenylcyclopropylamine) | Acts as a monoamine oxidase inhibitor |
| Phenelzine sulfate | Non-selective, irreversible monoamine oxidase inhibitor |
| Pargyline hydrochloride | Irreversible monoamine oxidase B inhibitor |
| Chlorpheniramine | Alkylamine antihistamine |
| Clemastine | Selective histamine h1 antagonist |
| Mebhydrolin naphthalenesulfonate | Histamine H₁-receptor antagonist |
| Dl-homatropine | Antagonist at muscarinic acetylcholine receptors |
| Oxyphenonium bromide | Antimuscarinic |
| Scopolamine | Acts as a competitive antagonist at muscarinic acetylcholine receptors |
| Telenzepine | Selective m1 antimuscarinic |
| Strychnine | Acts as an antagonist of glycine and acetylcholine receptors |
| Pralidoxime mesylate | Binds to organophosphate-inactivated acetylcholinesterase |
| Bromopride | Dopamine antagonist |
| Propiomazine maleate | Dopamine receptors (1, 2 and 4) antagonist |
| D-homocysteinesulfinic acid | Agonist at metabotropic glutamate receptors (MGLURS) |
| Zopiclone | Binds to α1, α2, α3 and α5 GABA_a_ benzodiazepine receptor complexes |
| Thiopental sodium | Acts on the GABA_a_ receptor |
| Eseroline | Acts as an opioid agonist |
| Bucetin | Acts as an analgesic and antipyretic |
| Phenylephrine hydrochloride | Selective α1-adrenergic receptor agonist |
| Urethane | Long-acting anesthetic with minimal cardiopulmonary depression |
| Lithium salicylate | Neuroactive lithium salt |
| Clobetasone butyrate | Glucocorticoid receptor agonist |
| Flunisolide | Glucocorticoid receptor agonist |
| Clobetasol propionate | Glucocorticoid receptor agonist |
| Epicatechin gallate (epigallocatechin (-)) | Antioxidant |
| Trans,trans-2,4-hexadien-1-ol | Antioxidant |
| Carnosine | Antioxidant |
| Choline magnesium trisalicylate | Nonsteroidal anti-inflammatory medications |
| Fosfosal (o-carboxyphenyl phosphate) | Salicylic acid derivative used in analgesic and anti-inflammatory therapy |
| Vitamin a palmitate (retinol palmitate) | Vitamin A |
| Retinyl palmitate | Synthetic alternate for retinyl acetate in vitamin A supplements |
| Regular iletin II (insulin injection, usp purified pork) | Insulin |
| Human insulin - ultralente - extended zinc suspension | Insulin |
| Human insulin - NPH - isophane suspension | Insulin |
| Norgestrel d(-) (levonorgestrel) | Progesterone receptor agonist |
| Drospirenone and ethinyl estradiol | Progesterone receptor agonist |
| Norethindrone acetate | Progesterone receptor agonist |
| Tibolone | Acts as an agonist mainly at estrogen receptors, with a preference for ER alpha |
| Spironolactone | Antagonist of the mineralocorticoid (or aldosterone) and androgen receptors |
| Iopanoic acid | Inhibits thyroid hormone release and peripheral conversion of thyroxine (T4) to triiodothyronine (T3) |
| 2-thiouracil | Inhibits thyroid activity by blocking thyroid peroxidase |
| Dinoprostone (prostaglandin e2) | Naturally occurring prostaglandin e2 (PGE2) |
| Sulfabenzamide | Antibacterial/antimicrobial |
| Pneumococcal 7-valent conjugate vaccine | Bacterial Vaccine |
| Linezolid | Bacterial protein synthesis inhibitor |
| Penicillin G sodium | Inhibits the formation of peptidoglycan cross-links in the bacterial cell wall |
| Gatifloxacin | Inhibits the bacterial enzymes DNA gyrase and topoisomerase iv |
| Methacycline hydrochloride | Inhibits bacterial protein synthesis |
| Cefuroxime axetil | Cephalosporin antibiotic |
| Cefmetazole | Cephalosporin antibiotic |
| Cefamandole | Cephalosporin antibiotic |
| Ristocetin sulfate | Antibiotic that belongs to the class III glycopeptides |
| Hydroquinidine HCl | Class I antiarrhythmic agent (Ia) in the heart |
| Practolol | Selective beta blocker that has been used in the emergency treatment of cardiac arrhythmias |
| N(g)-nitro-l-arginine | Inhibitor of nitric oxide synthase |
| Spermidine trihydrochloride | Inhibits neuronal nitric oxide synthase (nNOS). |
| L-arginine l-glutamate salt | Substrate of nitric oxide synthase |
| Triamterene | Blocks the epithelial sodium channel (ENAC) |
| Protoveratrine B | Na+ channel agonist |
| Metolazone | Inhibits the sodium-chloride symporter |
| Ibutilide fumarate | Acts on the slow sodium channel and promotes the influx of sodium through these slow channels |
| 3-formyl rifamycin | Interacts with membranes causes change in permeability to K+ and H+ in the mitochondrial membrane |
| Nigericin sodium | H+, k+ ionophore |
| Cloxyquin (5-chloro-8-hydroxy-quinoline) | Activator of the two-pore domain potassium channel TRESK |
| Enalapril maleate | Angiotensin-converting-enzyme (ACE) inhibitor |
| Fosinopril sodium | Angiotensin converting enzyme (ace) inhibitor |
| Adenosine 5'-(β,γ-imido)triphosphate tetralithium | A non-hydrolyzable ATP analog |
| Metformin (1,1-dimethylbiguanide hydrochloride) | Activates AMP-activated protein kinase (AMPK) |
| Zaprinast | Phosphodiesterase inhibitor, selective for subtypes PDE5, PDE6, PDE9 and PDE11 |
| Acetylphenylalanine | Amino acid derivative |
| N-(9-fluorenylmethoxycarbonyl)-l-Leucin | Amino acid derivative |
| L-aspartic acid | Amino acid |
| Lomofungin | Chelating agent for bivalent cations |
| Ferron (8-hydroxy 7 iodo 5 quinoline sulfonic acid) | Reagent for the extraction and spectrophotometric determination of Fe |
| Deferoxamine mesylate | Acts by binding free iron in the bloodstream and enhancing its elimination in the urine |
| Amsacrine | Intercalates DNA, inhibits topoisomerase ii |
| Homidium bromide (ethidium bromide) | Intercalates DNA |
| Benzo[a]pyrene | Intercalates DNA |
| Lomustine | Alkylates and cross-links DNA and RNA, thus inducing cytotoxicity |
| 2'-deoxyguanosine | Component of deoxyribonucleic acid |
| 2'-deoxycytidine | Component of deoxyribonucleic acid |
| Hirudin | Blood anticoagulant peptide |
| Mebrofenin ((3-bromo-2,4,6-trimethylphenylcarbamoyl) methyliminodiacetic acid) | Diagnostic aid 99mTc complex as diagnostic aid (radioactive imaging agent) |
| Potassium phosphate dibasic | Laxative |
| Sennoside B | Anthraquinone glucosides that are the laxative principles of senna. |
| Guaifenesin | Acts as an irritant to gastric vagal receptors |
| N-ethyldexoynojirimycin | Inhibitor of HIV cytopathicity |
| Formestane (4-androsten-4-ol-3,17-dione) | Type I, steroidal aromatase inhibitor |
| Oleic acid (cis-9-octadecenoic acid) | Monounsaturated omega-9 fatty acid |
| Meparfynol (methylpentynol, 3-methyl-1-pentyn-3-ol) | Initiator during the synthesis of propargyl-terminated polylactide by bulk ring-opening polymerization |
| Glycerol dimethacrylate | Adhesive and sealant formulations |
| Bi-lawsone (2,2-bi(3-hydroxy-1,4-naphthoquinone)) | Natural dye that reacts chemically with the keratin in skin and hair |
| Geneticin (G418) | Blocks polypeptide synthesis by inhibiting the elongation step in both prokaryotic and eukaryotic cells |
| Bismuth subcarbonate (bismuth carbonate oxide) | Antidiarrheal agent |
| Acetaldehyde | Precursor to acetic acid |
| Juniper tar | Anti-pruritic agent |
| 2-phenoxyethanol | Chemical preservative |
| Peppermint spirit | Therapeutic plant extract |
| Stannous octoate (tin(ii) 2-ethylhexanoate) | Polymerization initiator |
| Soy protein | Protein that is isolated from soybean. |
| Triethylene glycol diacetate | Thermoplastic acrylic coating as a reflow solvent |
| Flucytosine (5-fluorocytosine) | Converted into 5-fluorodeoxyuridinemonophosphate which inhibits fungal DNA synthesis |
| Acridine yellow | Fluorescent dye |
| O-phenanthroline | Inhibitor of metallopeptidases |
| Griseofulvin | Binds to tubulin, interfering with microtubule function, thus inhibiting mitosis |
| Thiourea | Chaotropic agent; strong denaturant. |
| Tomatine | Immune adjuvant in connection with certain protein antigens |
| Juglone | Inhibits the members of the parvulin PPIase family, antibiotic, anthelmintic |
| Coenzyme B-12 | Vitamin |
| Sulfamethazine | Induces CYP3A4 expression and acetylated by N-acetyltransferase |
| Tetracaine hydrochloride | Allosteric blocker of calcium release channel |
| Abscisic acid (cis,trans+/-) | Natural and active isomer of the abscission accelerating plant hormone |
| Crassin acetate | Immunosuppressant |
| Aesculin | Coumarin glucoside that naturally occurs in the horse chestnut |
| Diprotin A | Inhibitor of dipeptidyl aminopeptidase IV |
| Cyclocreatine | Protectant against inhibition of cardiac mitochondrial respiration. |
| Methoprene (s) | Mimics natural juvenile hormone of insects |
| Dinitolmide | Broad-spectrum anticoccidial drug |
| Cypromethin | Fast-acting neurotoxin in insects |
| Ciprofloxacin | Inhibits DNA gyrase, type II topoisomerase, and topoisomerase iv, enzymes |
| Stearoylcarnitine chloride | Protein kinase C inhibitor |
| Vinblastine sulfate | Binds tubulin, thereby inhibiting the assembly of microtubules |
| Oxantel | Inhibits fumarate reductase |
| 3,4-dichlorobenzylamine | n/a |

**Supplementary file 2. Outstanding Hit Calls**

The 131 compounds implicated in the primary screen but which remain to be further evaluated are listed here in subgroups according to their implicated pharmacological activity (shaded subsets). Subcategories included: Neuromodulators, glucocorticoids, and retinoids - also implicated by Tsuji et al., - as well as 11 other categories unique to our study. n/a: not applicable
